# CILAIR-Based Secretome Analysis of Obese Visceral and Subcutaneous Adipose Tissues Reveals Distinctive ECM Remodeling and Inflammation Mediators

**DOI:** 10.1038/srep12214

**Published:** 2015-07-22

**Authors:** Arturo Roca-Rivada, Susana Belen Bravo, Diego Pérez-Sotelo, Jana Alonso, Ana Isabel Castro, Iván Baamonde, Javier Baltar, Felipe F. Casanueva, María Pardo

**Affiliations:** 1 Grupo Obesidómica, Instituto de Investigación Sanitaria de Santiago (IDIS), Xerencia de Xestión Integrada de Santiago (SERGAS), Santiago de Compostela, Spain; 2 CIBER Fisiopatología Obesidad y Nutrición (CB06/03), Instituto de Salud Carlos III, Spain; 3 Unidad de Proteómica, Instituto de Investigación Sanitaria de Santiago de Compostela (IDIS), Santiago de Compostela, Spain; 4 Laboratorio de Endocrinología Molecular y Celular, Instituto de Investigación Sanitaria de Santiago (IDIS), Xerencia de Xestión Integrada de Santiago (SERGAS), Spain; 5 Servicio de Cirugía General, Xerencia de Xestión Integrada de Santiago (SERGAS), Santiago de Compostela, Spain

## Abstract

In the context of obesity, strong evidences support a distinctive pathological contribution of adipose tissue depending on its anatomical site of accumulation. Therefore, subcutaneous adipose tissue (SAT) has been lately considered metabolically benign compared to visceral fat (VAT), whose location is associated to the risk of developing cardiovascular disease, insulin resistance, and other associated comorbidities. Under the above situation, the chronic local inflammation that characterizes obese adipose tissue, has acquired a major role on the pathogenesis of obesity. In this work, we have analyzed for the first time human obese VAT and SAT secretomes using an improved quantitative proteomic approach for the study of tissue secretomes, Comparison of Isotope-Labeled Amino acid Incorporation Rates (CILAIR). The use of double isotope-labeling-CILAIR approach to analyze VAT and SAT secretomes allowed the identification of location-specific secreted proteins and its differential secretion. Additionally to the very high percentage of identified proteins previously implicated in obesity or in its comorbidities, this approach was revealed as a useful tool for the study of the obese adipose tissue microenvironment including extracellular matrix (ECM) remodeling and inflammatory status. The results herein presented reinforce the fact that VAT and SAT depots have distinct features and contribute differentially to metabolic disease.

Obesity has become one of the largest medical problems in the modern society reaching pandemic proportions as it is actually affecting more than 500 million adults worldwide^1^. The principal concern about obesity is its association with different metabolic diseases such as type 2 diabetes, dyslipidemia, metabolic syndrome and even several types of cancer^2^,^3^,^4^,^5^. According to the estimated progression of obesity in the next decades, without a solution, the effects of this disease in the society will be dramatically expensive at both social and economical level^6^,^7^.

Obesity comprise an excessive increase in the energy storage of the organism through the deregulation of white adipose tissue (WAT) energy balance^8^. Despite that during the last 20 years the knowledge about the endocrine role of adipose tissue has developed enormously, especially in obesity situations^9^, obesity disease is far to have a solution due to the complexity of the adipose tissue itself and its relationship with other endocrine organs at peripheral and central level^10^,^11^. Part of this complexity is related to the different cell types that compose adipose tissue (adipocytes and cells from the stromal vascular fraction) and their interaction. This circumstance makes, as a result, the study of single cell type cultures inappropriate showing normally a limited view of their behavior in the organism^12^. Interactions between cells from the stromal fraction and adipocytes are necessary for the physiological functionality of adipose tissue, and deregulation of this cross-talk is regarded as an important mechanism leading to insulin resistance and type 2 diabetes^13^; for example, high levels of adipocyte-secreted protein fetuin-A have showed to promote macrophage infiltration contributing to the obesity-related inflammation^14^. Moreover, it was shown that the differentiation and proliferation capacity of adipose tissue is influenced by the cross-talk between adipocytes and macrophages from the stromal vascular fraction^15^.

Considering that it is currently a fact that WAT physiology, morphology and functionality depends on its anatomical location, in those studies involving whole adipose tissue, the source of origin of the selected sample becomes an important issue^16^,^17^,^18^. The excessive amount or the malfunction of certain adipose depots such as visceral fat has been linked to the development of several metabolic diseases^19^; thus, the specific origin of the adipose tissue in future studies is a critical point that needs to be well appointed.

The in-depth investigation about the specific endocrine regulation of the different adipose depots, focusing in obesity situations, is an essential step to understand the development of the metabolic diseases related to the amount and deregulation of WAT in order to establish new therapies in the future to prevent the appearance of these diseases^20^. For such studies, proteomic techniques have proven to be very useful in the identification of new adipokines and the characterization of their expression and secretion by WAT^12^,^21^. The emerge in the recent years of more accurate and precise techniques based on quantitative methods such as Stable Isotope Labeling by Amino acids in Cell culture (SILAC)^22^, have generated a great improvement in all areas of cell biology studies involving novel protein identification and characterization^23^. To make SILAC methodology compatible with adipose tissue culture, Roelofsen and collaborators developed a variation method defined as Comparison of Isotope-Labeled Amino acid Incorporation Rates (CILAIR) to analyze newly synthesized secreted proteins at a fixed culture period^24^. In this approach, the proteins containing label are synthesized by the tissue avoiding the presence of contaminant proteins from the serum or damaged cells. This unique CILAIR study analyzing the effect of insulin on human healthy omental tissue explants proved to render valuable information^24^.

In the present work we show that the CILAIR approach can be enriched by double amino acid labeling ([^13^C6]-Lys and [^13^C6, ^15^N4]-Arg), becoming a powerful tool to study protein secretion from human adipose tissue samples, and appropriate to compare depots from the same patient in the same conditions. This is the first CILAIR study analyzing obese adipose tissue from visceral and subcutaneous origin. The presented data shows that the visceral and the subcutaneous adipose tissue depots present different secretory profiles in obesity situations, reinforcing the differentiated role of each depot in the development of obesity and its comorbidities. The establishment of this methodology opens new perspectives in the development of personal therapies in the future.

## Materials and Methods

### Human adipose tissue acquisition

Human adipose tissue for the CILAIR study was obtained from three non-diabetic obese patients (body mass index >35) with metabolic syndrome who underwent laparoscopic gastrectomy surgery. Additionally, samples from three healthy (body mass index <30) voluntary subjects undergoing abdominal surgery and three extra samples from obese non-diabetic subjects were collected for validation purposes by immunoblotting. A written informed consent was obtained from all the subjects. All procedures were approved by the Clinical Ethical Committee of Galicia (CEIC), Spain under the code number 2013/425. All experiments were carried out in accordance with the approved guidelines and regulations. The visceral fat (VAT) was extracted from the hypogastric region around the internal organs, and the subcutaneous fat (SAT) from the mesogastric region. The tissues were transported from the operating room to the laboratory in sterile KRH buffer with penicillin (100 U/ml) and streptomycin (100 μg/ml).

### CILAIR

The adipose tissue culture and labeling protocol was performed as previously described by Roelofsen and collaborators with some variations^24^. Briefly adipose tissue explants were processed to eliminate any contaminants under a laminar flow hood using sterile equipment and transferred to a Petri dish to perform an intensive wash in 20 ml of PBS that was repeated several times with gently shaken for a short period. The tissue pieces were transferred to a tube containing 25 ml of PBS and centrifuged for 5 min at 1800 rpm at room temperature to remove red blood cells and debris. The tissue was then removed from the tube, and the weight was determined. 1.2 g pieces of each tissue type (VAT or SAT) were incubated at 37 °C and 5% CO2 in 5 ml/tissue piece/well of lysine and arginine-free DMEM medium (Silantes, München, Germany) supplemented with 50 mg/ml penicilin-streptomicin. The medium was renewed after 1, 18, 22, and 26 h. After the last wash (time point, 26 h), all dishes received fresh DMEM medium (3 ml) containing 70 mg/liter heavy-labeled lysine (L-[^13^C6]-Lys, Silantes, Germany) and 70 mg/liter heavy-labeled arginine (L-[^13^C6,^15^N4]-Arg, Silantes, Germany).Tissues were maintained in culture for an additional 72 h to allow incorporation of the label into newly synthesized proteins. Thereafter media were collected and stored at −80 °C until analysis.

### Protein Identification by LC-MS/MS

Three milliliters of adipose tissue culture medium were concentrated by ultrafiltration (Amicon Ultra 5 ml 3 kDa, Millipore, MA, USA). This concentrated sample was further concentrated by ultrafiltration (Amicon ultra 0,5 ml 3 kDa, Millipore) to a final volume of approximately 40 μl. Proteins present in the concentrated adipose tissue medium sample were quantified (RC DC Protein Assay, BioRad Lab, CA, USA) and an equal amount of protein (200 μg) from all conditions were loaded in a 10% SDS-PAGE gel. The run was stopped as soon as the front entered 3 mm into the resolving gel^25^. The protein band was visualized by Sypro-Ruby fluorescent staining (Lonza, Switzerland) excised and submitted for in-gel manual tryptic digestion following standard procedure with minor modification^26^. The bands were washed in 50 mM ammonium bicarbonate with 50% MeOH (HPLC grade, Scharlau, Barcelona, Spain) and dehydrated in acetonitrile (ACN) (HPLC grade, Scharlau, Barcelona, Spain), followed by in-gel reduction with 10 mM dithiothreitol (Sigma-Aldrich, MO, USA) in 50 mM ammonium bicarbonate (Sigma-Aldrich) (30 min at 56 °C) and alkylated with 55 mM of iodoacetamide (Sigma- Aldrich) in 50 mM ammonium bicarbonate (20 min at room temperature in the dark). Gel pieces were subsequently washed with 50 mM ammonium bicarbonate in 50% MeOH (HPLC grade, Scharlau, Barcelona, Spain), and dehydrated with pure CAN. Tryptic digestion was performed by the addition of 6 μl of 20 ng/μl of modified trypsin (Promega, CA, USA) in 20 mM ammonium bicarbonate, and gel pieces were allowed to rehydrate on ice for 20 min. Digestion was carried out overnight at 37 °C. Peptides were extracted thrice by 20 min incubation in 40 μL of 60% acetonitrile in 0.5% HCOOH. The resulting peptide extracts were pooled, concentrated in a SpeedVac (Thermo Scientific, USA) and stored at −20 °C.

Separation of the resulting tryptic peptide mixtures was performed by nanoscale reversed-phase LC-MS/MS. The nanoLC Ultra 1D plus*, (EKsigent,* ABSciex, MA, USA) was coupled to a MALDI-spotter (Eksigent, ABSciex). Peptides mixtures were re-dissolved in 0.1% formic acid, 2% ACN and injected into the trapping column (ChromXP nanoLC Trap column 350 μm id × 0.5 mm, ChromXP C18 3 μm 120 Å, ABSciex) at a flow rate of 10 μl/min (0.1% formic acid 2% ACN). After 15 min the trapped peptides were separated in a nanocolumn (ChromXP nanoLC column 75 μm id × 15 cm, ChromXP C18 3 um 120 Å, ABSciex) at a flow rate of 300 nl/min in a linear gradient elution from 95% A (0.1% formic acid 2% ACN) to 60% B (90% ACN, 0.1% FA) in 80 min followed by an increase up to 95% B in 5 min. The eluting peptides were mixed with a matrix solution, consisting of 3 mg alpha-cyano-4-hydroxycinnamic acid (α-CHCA) dissolved in 1 mL of 50% ACN in 0.1% trifluoroacetic acid, and 10 fmol/μl angiotensin I (Sigma Aldrich) (as internal standard) and spotted onto a Opti-TOF LC/MALDI insert (ABSciex, USA) with a speed of one spot per 12 seconds.

### Mass Spectrometric Analysis

Mass spectrometry analysis was made using a 4800 MALDI-TOF/TOF analyzer (ABSciex). MS spectra were acquired in reflector positive-ion mode with a Nd:YAG, 355 nm wavelength laser, averaging 1000 laser shots and using the peak (1296.685 Da) of angiotensin I as internal calibration. All MS/MS spectra were performed by selecting the precursors with a relative resolution of 300 (FWHM) and metastable suppression.

### Data analysis

Peptide and protein identification were performed using the Protein Pilot software v 4.0.80.85 (ABSciex) with Paragon Algorithm. MS/MS data was searched against the UniProt/Swiss-Prot database of protein sequences (July 2013; Swiss-Prot, Geneva, Switzerland 547357 entries). Searches were restricted to human taxonomy allowing carbamidomethyl cysteine as a fixed modification and oxidized methionine as variable modification. Heavy-labeling of Lys and Arg were selected as follows: Δmass +6 and Δmass +10, respectively. Both the precursor mass tolerance and the MS/MS tolerance were set at 30 ppm and 0.35 Da, respectively, allowing 1 missed tryptic cleavage site.

The software automatically detects the heavy/light peak pairs and calculates the heavy/light ratios based on the peak areas. The automatic correction for mixing errors (normalization to median) when performing CILAIR was turned off. Only proteins with a threshold >95% confidence (>1.3 unused score) were considered as positive hits.

Classification of identified proteins as potentially secreted was performed by SecretomeP 2.0 server (Centre for Biological Sequence Analysis)^27^. For each input sequence (FASTA format) the server predicts the possibility of non-classical secretion giving high score to proteins entering the classical secretory pathway (those proteins with signal peptide). Non-classically secreted proteins should obtain an NN-score exceeding the normal threshold of 0.5.

Functional analysis was performed by FunRich open access software (Functional Enrichment analysis tool) for functional enrichment and interaction network analysis (http://funrich.org/index.html). For statistics, FunRich use hypergeometric test, BH and Bonferroni.

### Immunoblotting

For the one dimensional (1-DE) western blot 50 μg, of secreted proteins from obese and healthy independent subjects were resuspended in Laemmli sample buffer and loaded in 12% SDS-PAGE gels. In the case of two-dimensional (2-DE) western blots, 80 μg of secreted proteins were taken into a final volume of 120 μl in 2-DE sample buffer containing 5 M urea, 2 M thiourea, 2 mM tributylphosphine, 65 mM DTT, 65 mM CHAPS and 0.15 M NDSB-256. Ampholytes were added to the sample at 0.1% servalyte 3–10, 0.05% servalyte 2–4 and 9–11 (SERVA, Heidelberg, Germany). 3–10 NL 7 cm IPG strips (BioRad, CA) were actively rehydrated in the sample and IEF was carried out in a Protean IEF cell following the manufacturer protocol (BioRad, CA). Following focusing, the IPG strips were immediately equilibrated for 20 min in 4 M urea, 2 mM thiourea, 12 mM DTT, 50 mM Tris pH 6.8, 2%SDS, 30% glycerol. The IPG strips were placed on top of the second dimension gels (15% SDS-PAGE) and embedded with 1% melted agarose. Both 1-DE and 2-DE gels were electroblotted onto nitrocellulose membranes. Equal loading was confirmed by membrane staining with Ponceau S (Sigma-Aldrich; St Louis, MO). The membranes were probed successively with primary antibodies and alkaline phosphatase-labeled secondary antibodies (Thermo Scientific, MA). Specific antigen-antibody binding was visualized using a chemiluminescence method according to the manufacturer (ECL Western Blotting Substrate, Thermo Scientific, MA). In the 2-DE western blot, a single radiography film was used for the four experimental conditions. Primary anti-C3 (B-9) and anti-TIMP-1, were purchased from Sta. Cruz Biotechnology (CA, USA).

### Statistical analysis

For this study we used samples of three patients that were cultured in two or more plates to make at least two replicates of each sample type for patient. The statistical significance of the differences between mean values was determined using a two-tailed t-test, considering P ≤ 0.05 significant. In the proteomic analysis, normalization tools and the statistical package from Protein Pilot software were employed (ABSciex). We considered statistically significant only those changes with P ≤ 0.05. In the immunoblotting studies, data analyses were conducted using GraphPad Prism 5 software with a Mann-Whitney U test in which *p < 0.05 and **p < 0.01 were considered significant and very significant, respectively.

## Results

### Double isotope-labeled amino acid incorporation for VAT and SAT secretome analysis by CILAIR

**Figure 1:**
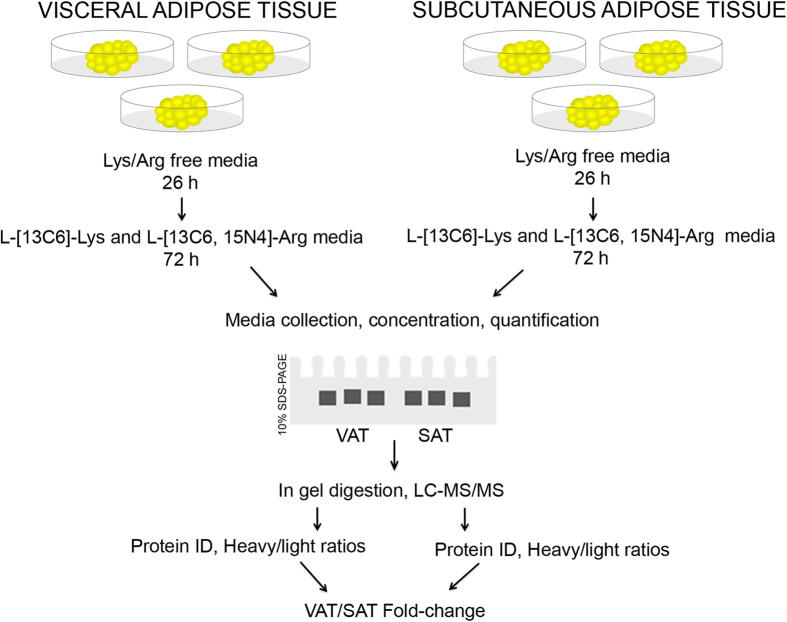
Experimental workflow. VAT and SAT explants from 3 independent obese patients were extensively washed and minced in equal weight fragments. After incubation with Lys/Arg free media for 26 hours, samples were labeled with 70 mg/liter of L-[^13^C6]-Lys and L-[^13^C6, ^15^N4]-Arg for 72 hours. Labeled secretomes were collected, concentrated and quantified; then, equal amount of proteins were loaded in a 10% SDS-PAGE gel and run until a single band entered the resolving gel 3  mm. This unique band was processed for in gel digestion followed by LC-MS/MS. Proteins were identified and heavy/light ratios were determined resulting in three different data sets for VAT and SAT. Overlap data from VAT and SAT of the same patient was determined and the fold-change values calculated for each protein by dividing their VAT heavy/light ratios in relation to SAT.

Human visceral (VAT) and subcutaneous (SAT) adipose tissue sample explants were obtained from three obese individuals (Patients 1, 2 & 3) during bariatric surgery as described in the materials and methods section. Depending on the size of the extracted sample, the experiment was performed in at least 2 replicates using equal-sized tissue pieces from the same patient. Secretomes from SAT and VAT explants were collected after *in vitro* incubation with L-[^13^C6]-Lys and L-[^13^C6,^15^N4]-Arg for 72 hours and processed for CILAIR analysis (Fig. 1).

**Figure 2:**
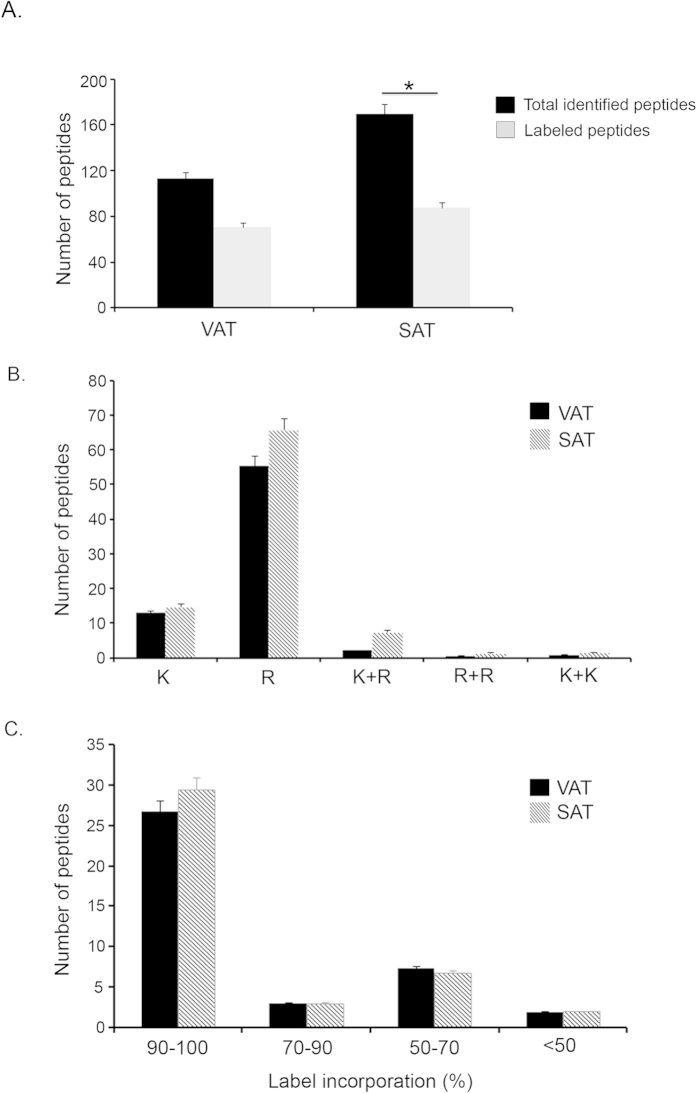
Double isotope-labeled amino acid incorporation in secretomes from VAT and SAT explants. The number of total labeled peptides in VAT and SAT in relation to total identified peptides after a 72 hours labeling period is represented in histograms (**A**). The number of peptides labeled with Lys (K), Arg (R), or different combinations of isotope-labeled amino acids (K+R, R+R and K+K) is shown for VAT and SAT (**B**). Labeled amino acid incorporation efficiency is represented as percentage of labeled peptides in VAT and SAT.

In order to detect any possible difference on the metabolic incorporation labeling rate between VAT and SAT, the quantity of labeled peptides was measured in both secretomes. This data proved that there was a good correlation between the total of peptides identified and the ones labeled. Thus, after 72 hours, the number of labeled peptides was about 60% of the total identified peptides in VAT and 50% in SAT (Fig. 2A). Additionally, the analysis of the amount of labeled peptides revealed that arginine was incorporated at a higher degree than lysine in both tissue types (Fig. 2B). As expected, the number of peptides containing different combinations of both amino acids were detected in lesser extends (Fig. 2B).

The analysis of heavy amino acids total incorporation tested by quantifying CILAIR ratios of all labeled identified peptides for each protein showed incorporation efficiency above 90% on average in both tissues (Fig. 2C).

### Double labeling incorporation analysis in VAT and SAT

The liquid chromatography followed by mass spectrometry analysis of VAT and SAT secretomes allowed the identification of 47 different labeled proteins in at least 60% of VAT replicates (100% classified as secreted proteins: 98% containing signal peptide), and 42 in SAT (100% classified as secreted proteins containing signal peptide) (Tables 1, 2, 3). 39 proteins were common to both adipose depots (Table 1); from those 12 proteins were significantly over-secreted in VAT compared to SAT (p < 0.05) 10 over-secreted in SAT (p < 0.05), and 17 showed no changes (Table 1). Among significant differences, the top 5 proteins according to the H/L ratio in VAT were basement membrane-specific heparin sulfate proteoglycan core protein (HSPG2), laminin subunit alpha-4 (LAMA4), collagen alpha-3 (VI) chain (COL6A3), versican core protein (VCAN) and transforming growth factor-beta-induced protein ig-h3 (TGFBI). In SAT, inter-alpha-trypsin inhibitor heavy chain H5 (ITIH5), SPARC-like protein 1 (SPARCL1), metalloproteinase inhibitor 1 (TIIMP1), coiled-coil domain-containing protein 80 (CCDC80), and thrombospondin-1(THBS2) showed the highest fold-change compared to VAT.

Moreover, COL18A1, COL4A2, periostin, nidogen-2, galectin-3-binding protein, cystatin C, complement factor B and pigment epithelium-derived factor (SERPINF1) were only detected in VAT (Table 2); and SERPINA 1, galectin-3 and MMP3 proteins were exclusively labeled in SAT (Table 3).

**Figure 3:**
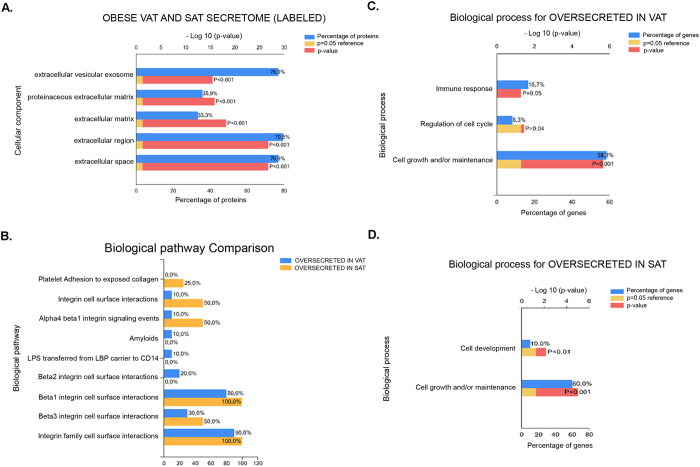
Functional analysis. All labeled proteins identified in obese VAT and SAT secretomes were submitted to FunRich functional analysis tool as indicated in the methods section. Cellular component classification of all VAT and SAT secreted proteins are shown (**A**). Comparison of proteins oversecreted in VAT vs SAT regarding biological pathways classification is illustrated (**B**). The categorization in terms of biological process of oversecreted proteins in VAT (**C**) and SAT (**D**) independently is also represented.

Functional classification of all labeled proteins in the three obese patients confirmed the extracellular/secreted nature of the identified proteins being a high percentage classified as from the extracellular region (79.5%), extracellular space (76.9%) or extracellular vesicular exosome (76.9%) (Fig. 3A). On the other hand, the comparison of oversecreted proteins in VAT and SAT showed their role in distinct biological pathways (Fig. 3B). Despite that more than 50% of oversecreted proteins were classified as implicated in cell growth and/or maintenance in both depots, VAT secretome was characterized by proteins involved in immune response and regulation of the cell cycle, and SAT by cell development (Fig. 3C,D).

**Figure 4:**
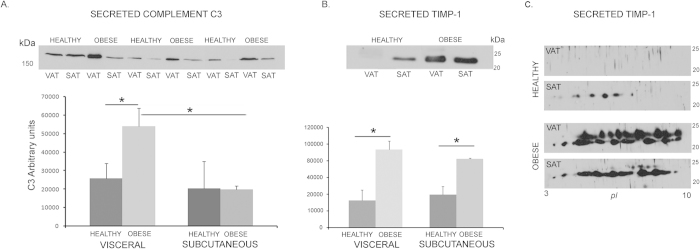
Validation of selected proteins. Independent obese and healthy secretome samples from VAT and SAT depots were assayed by immunoblotting. C3 protein detection cropped image and histogram graph from band quantification is shown (**A**); TIMP-1 representative cropped image of 1-DE immunodetection of healthy and obese VAT and SAT secretomes (**B**); and 2-DE representative cropped western blots showing post-translational variants secreted by VAT and SAT from healthy and obese individuals (**C**). VAT: visceral adipose tissue; SAT: subcutaneous adipose tissue. Statistical significance is represented as *p < 0.05.

Protein secretion validation was performed selecting C3 as oversecreted in VAT and TIMP-1 as oversecreted in SAT (Fig. 4). Immunodetection of C3 in independent human healthy and obese secretomes from VAT and SAT explants confirmed the oversecretion of C3 by visceral adipose tissue compared to subcutaneous in obesity (p = 0.05) and revealed the elevated secretion of obese VAT in relation to healthy VAT tissue (p = 0.06) (Fig. 4A). Regarding TIMP-1, although the obese secretomes were characterized by higher levels of this protein compared to healthy adipose tissues (p < 0.05), no significant differences were found among obese VAT and SAT by 1-DE immunoblotting (p = 0.35) (Fig. 4B); however, 2-DE western blot revealed important post-translational differences in secreted TIMP-1 by obese VAT compared to SAT (Fig. 4C).

### Non-labeled secreted proteins by VAT and SAT

In view of the fact that the labeling incubation time is very limited in the CILAIR approach, the non-labeled secreted proteins in VAT (136 proteins classified as secreted) and SAT (64 proteins classified as secreted) were also considered and shown in Supplementary Table 1. Classical known adipokines such as adiponectin and leptin, and other proteins of interest such as CD44 antigen, follistatin-related protein1, insulin-like growth factor-binding protein 4 and 7 were detected in the VAT secretome from obese individuals.

## Discussion

In the present study we perform for the first time a double heavy isotope-labeling of visceral and subcutaneous human obese adipose tissues with the aim to identify tissue specific secreted proteins. By analyzing the incorporation rate of [^13^C6]-Lys and [^13^C6, ^15^N4]-Arg after a defined period of time, this approach allowed us the comparison of VAT and SAT secretomes from the same obese patient. This analysis reinforced previous studies showing a differential secretion pattern of VAT and SAT depots in humans and its alteration in situations of nutrient excess such as obesity. Altogether, this work highlights the role of ECM remodeling and inflammation in human obese adipose tissue.

The use of global transcriptome profiling together with bioinformatics tools and the direct secretome analysis of cell cultures and tissues to detect new secreted proteins has been validated for the study of the human fat cell secretome^21^,^28^; however, these approaches show several limitations. In one hand mRNA expression pattern is not always representative of real protein secretion dynamics and do not reveals post-translational modifications of functional importance. In the other hand, secretome analysis, especially from tissues, is frequently contaminated with a high percentage of intracellular proteins caused by cell disruption inherent to this type of assay or with proteins from irrigating blood^20^. These disadvantages are solved in the present work by incorporating heavy-labeled amino acids into newly synthesized secreted proteins^29^. Precisely, a modified SILAC approach based on Comparison of Isotope-Labeled Amino acid Incorporation Rates (CILAIR) has been selected and improved^24^.

A previous CILAIR analysis of a healthy human adipose tissue secretome with or without insulin treatment showed that this methodology allows the quantitative assessment of changes in protein secretion without the need of 100% label incorporation^24^. Taking into account that peptides end in Lys and Arg after tryptic digestion, our approach, based on this previous work, introduces a second isotope-labeled amino acid ([^13^C6, ^15^N4]-Arg) for the study of fat secretomes in order to make a better quantification of labeled proteins. In that regard, the amount of Arg used in our study was not very high compared to standard procedures to prevent possible conversions of arginine to proline. Interestingly, we found that at equal quantities of labeled Arg and Lys, Arg was incorporated at a higher rate than Lys in both VAT and SAT explants in all the analyzed patients; this fact implies an improvement of the methodology for adipose tissue secretome analysis. However, the number of labeled proteins in the present work showed to be limited compared to the previous work by Roelofsen and collaborators^24^. This might be due to methodological differences regarding the previous SDS-PAGE fractionation in 25 bands compared to our single band analysis. Nevertheless, on the contrary to the previous work, we found that almost 100% of the labeled proteins were classified as secreted by signal peptide. In addition, functional analysis of all labeled proteins showed that a high percentage of the labeled proteins corresponded to the extracellular space, extracellular region and extracellular vesicular exosome. Interestingly, the number of labeled peptides compared to the total peptides identified was lower in SAT secretomes, which might suggest lesser metabolic activity of this tissue compared to VAT. In this regard, the number of non-labeled secreted proteins identified in VAT was much higher than SAT.

With reference to the labeled proteins detected exclusively in the VAT secretome, it has to be highlighted the extracellular matrix organization proteins cystatin-C (CST3) and COL4A2; the acute inflammatory response implicated protein complement factor B (CFB); and galectin-3-binding protein (LGALS3BP), nidogen-2 and COL18A1 classified as implicated in biological adhesion. The former were all previously found expressed in human primary adipocytes and up-regulated at mRNA level in adipose tissue of obese subjects^28^. Additionally, it is important to emphasize the detection of labeled periostin, a crucial extracellular remodeling factor in inflammatory microenvironments, only described in VAT secretomes^30^. In relation to PEDF, it was not surprising to find this protein as being one of the most abundant proteins secreted by adipose tissue^31^. Interestingly, growing evidence suggests that this factor is associated with visceral adiposity^32^,^33^. Moreover, previous work suggests that PEDF secreted by adipocytes may contribute to the onset and maintenance of chronic inflammation in obesity, and may be a therapeutic target in ameliorating insulin resistance^34^.

In relation to proteins exclusively labeled in SAT secretome, it is worth to highlight galectin-3. Precisely, in adipose tissue galectin-3 is synthesized by adipocytes and macrophages, and in accordance to our results, it was shown to be increased in subcutaneous fat of obese mice compared to VAT^35^,^36^,^37^. In relation to stromelysin 1 (MMP-3), it was shown that macrophage accumulation in adipose tissue has a central role in stimulating MMP1 and MMP3 production by preadipocytes, which might be partially mediated by IL-1β via activation of the MAPK and NF-κB signaling pathways^38^. Concerning the alpha-1-antitrypsin (SERPINA1), implicated in the acute inflammatory response, it has been recently nominated as a novel biomarker for obesity in humans^39^, and identified as up-regulated in obese adipose tissue^28^.

Focusing on the common identified proteins to both VAT and SAT, significant labeling VAT towards SAT ratios showed the over-secretion in VAT of different extracellular matrix constituents: basement membrane specific heparin sulfate proteoglycan core protein (HSPG2), three different types of collagen VI (COLA61, 2 and 3), two types of laminins (LAMA4 and LAMC1), versican core protein, transforming growth factor-beta-induced protein ig-h3 (TGFBI) and cathepsin L1. The discovery of COL6A over-secretion in VAT and not in SAT in our work is crucial due to its dysfunctional role in obesity contributing to the development of metabolic syndrome^40^. Collagen VI is ubiquitously expressed throughout connective tissues; however, adipose tissue is the most abundant source of this ECM protein^41^ and several evidences indicate its implication in obesity-related adipose tissue fibrosis promoting inflammation and insulin resistance^40^,^42^. Other proteins such as complement C3 implicated in acute inflammatory response and the macrophage marker CD14 were also detected only in VAT secretomes reinforcing the inflammatory status of visceral adipose tissue in obese individuals.

On the other side, SAT obese secretome was characterized by the over-secretion of a different set of ECM proteins compared to VAT. It is noteworthy to highlight the presence of the angiogenesis regulators thrombospondin1 and 2 (TSP-1, 2). TSP-1 is implicated in adiposity and metabolic dysfunction in diet-induced obesity enhancing adipose inflammation and stimulating adipocyte proliferation^43^,^44^,^45^; on the contrary, anti-angiogenic and anti-proliferative properties have been attributed to TSP-2^46^. Moreover, SAT secretome was characterized by collagen I type (COL1A1), recently associated to SAT^47^; metalloproteinase inhibitor 1 (TIMP1), SPARC-like protein 1 (SPARCL1), chitinase-3-like protein 1 (YKL-40), coiled-coil domain-containing protein 80 (CCDC80) and inter-alpha-trypsin inhibitor heavy chain H5 (ITIH5). TIMP-1 is a matrix metalloproteinase crucial for the extracellular reorganization which has been described as a negative regulator of adipogenesis; *in vivo*, TIMP1 leads to enlargement of adipocytes in state of overnutrition which might be responsible of systemic fatty acid overload, hepatic lipid accumulation and insulin resistance in TIMP-1 injected mice^48^. CCDC80 is a component of the extracellular matrix able to bind various extracellular matrix proteins and promote cell adhesion^49^. Loss of CCDC80 negatively modulates glucose homeostasis in diet-induced obese mice^50^. YKL-40 is secreted by activated macrophages and neutrophils and expressed in a broad spectrum of inflammatory conditions and cancers^51^. It was defined as a BMI-independent marker of type 2 diabetes and elevated in morbidly obese patients^52^,^53^,^54^. In relation to our study, YKL-40 was previously shown to be secreted by VAT at elevated levels in obesity-associated type 2 diabetes; its role in SAT was however not previously characterized in the bibliography to the best of our knowledge^55^. ITIH-5 expression in human adipose tissue has been very recently discovered by gene expression profile data. Interestingly, resembling our results, Anvenden and collaborators detected that ITIH-5 mRNA expression is abundant in human adipose tissue, and higher in subcutaneous compared to omental; besides, they describe higher expression levels in SAT than in VAT from obese subjects^56^. An *in silico* study of 347 adipokines from a human secretome by these same authors stands out ITIH-5 for further analysis as its coding gene scored among the highest in the analysis, being elevated in obesity^28^. Finally, it should be mentioned the angiopoietin-related protein 2 (ANGPTL2) that was described as key adipocyte-derived inflammatory mediator that links obesity to systemic insulin resistance^57^. The potential role of other identified proteins such as procollagen C-endopeptidase enhancer 1 and SPARC-like protein 1 in SAT remains elusive.

Interestingly, the functional classification according to the biological process of oversecreted proteins in obese VAT and SAT showed the secretion of proteins implicated in the immune response and the regulation of the cell cycle in VAT and in cell development in SAT. Further, some of our results parallels a previous report performed in healthy mice with CILAIR followed by N-glycosylated proteins enrichment. Despite is not comparable to our work, a distinct secretion pattern in visceral epididymal fat compared to inguinal subcutaneous fat is described^58^.

The limitations to our study comprise the impossibility to normalize the number of cells for each adipose depot taking also into account that we are culturing adipocytes and all the components of the stromal-vascular fraction; discerning the exact amount of each cell type is impracticable in this experimental setting. Additionally, regarding the validation of identified proteins it has to be considered that CILAIR detects changes in secretion of proteins synthesized *de novo*; when performing validation studies by western blot in the whole secretome, labeled and non-labeled contaminant proteins are present and therefore the validation results using this methodology has to be taken cautiously. However, the validation study for representative proteins in the present work reaffirms the CILAIR results for C3 and to a lesser extend for TIMP-1 due to the abundant post-traslational modifications which have been previously described in the literature^59^. Our studies cannot determine which TIMP-1 isoform was differentially secreted in SAT over VAT in the performed analysis.

The presence of adiponectin and leptin in the non-labeled identified proteins in this work indicates either that this experimental approach, characterized by limited label incorporation time (72 h), was unable to label important secreted adipokines, or that we were unable to detect them among the bulk of labeled peptides. The constitutive and chronic secretion of inflammation-related proteins in obese adipose tissue may eclipse the detection of some of the less abundant adipokines or even affect the secretion pattern, especially in situations of obesity^60^. The introduction of a second isotope-labeled amino acid in the experiment might require longer incubation periods. Further, the fractionation of the secretome sample in more than one SDS-PAGE band might probably override this issue. Still, among non-labeled there were several proteins of interest like CD44 antigen, follistatin-related protein 1 and insulin-like growth factor-binding protein 4 and 7.

In this study the majority of the identified secreted proteins were related with the extracellular matrix remodeling, including structural compounds, remodeling proteases and their inhibitors, and mediators of inflammation. These characteristic type of secreted proteins are in accordance with the described response of adipose tissue to nutrient excess where adipocyte undergoes hypertrophy and hyperplasia, followed by increased angiogenesis, immune cell infiltration, extracellular matrix (ECM) overproduction and the increased production of proinflammatory adipokines^61^. Precisely, the extracellular matrix components are a key factor in the development of the adipose tissue in obesity, and their specific composition seems to be directly implicated in the functionality of this tissue depending on their location, regulating processes like the capacity of expansion or their capacity to mobilize lipids^47^,^62^,^63^.

As final conclusion we prove the valuable use of double isotope-labeling-CILAIR approach to analyze VAT and SAT pathological secretomes and more precisely for the study of obese adipose tissue extracellular matrix remodeling and inflammatory status. Importantly, all labeled proteins were classified as secreted, and moreover, a very high percentage of these proteins were in one way or another previously implicated in obesity or in its comorbidities. The results herein presented reinforce the fact that visceral and subcutaneous adipose tissue depots have distinct features and contribute differentially to metabolic disease.

## Additional Information

**How to cite this article**: Roca-Rivada, A. *et al.* CILAIR-Based Secretome Analysis of Obese Visceral and Subcutaneous Adipose Tissues Reveals Distinctive ECM Remodeling and Inflammation Mediators. *Sci. Rep.* **5**, 12214; doi: 10.1038/srep12214 (2015).

## Supplementary Material

Supplementary Information

## Figures and Tables

**Table 1:**
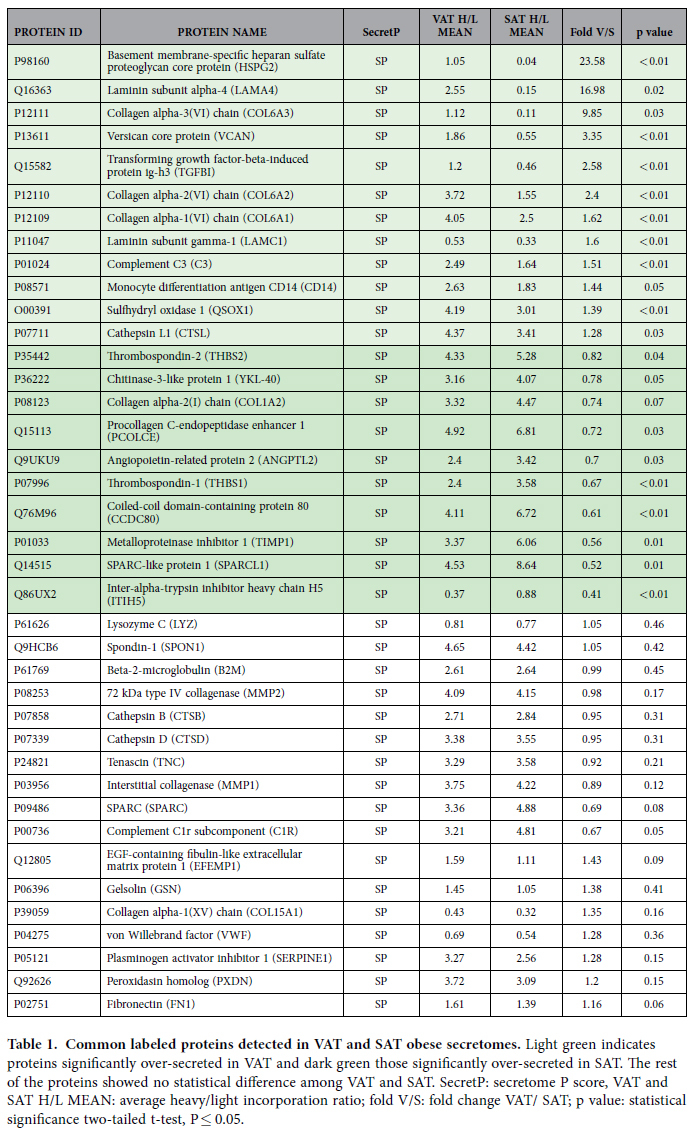
Common labeled proteins detected in VAT and SAT obese secretomes.

**Table 2:** Labeled proteins detected exclusively in VAT obese secretome.

**ONLY VAT**			
**PROTEIN ID**	**PROTEIN NAME**	**SecretP**	**MEAN**
P39060	Collagen alpha-1(XVIII) chain (COL18A1)	SP	0.31
P08572	Collagen alpha-2(IV) chain (COL4A2)	SP	1.08
P00751	Complement factor B (CFB)	SP	3.74
P01034	Cystatin-C (CST3)	SP	3.10
Q08380	Galectin-3-binding protein (LGALS3BP)	SP	2.84
Q14112	Nidogen-2 (NID2)	SP	0.99
Q15063	Periostin (POSTN)	SP	0.98
P36955	Pigment epithelium-derived factor (SERPINF1)	SP	5.61

SecretP: secretome P score, SP: Signal Peptide, MEAN: average heavy/light incorporation ratio.

**Table 3:** Labeled proteins detected exclusively in SAT obese secretome.

**ONLY SAT**			
**PROTEIN ID**	**PROTEIN NAME**	**SecretP**	**MEAN**
P01009	Alpha-1-antitrypsin (SERPINA 1)	SP	0.22
P17931	Galectin-3 (LGALS3)	0.77	1.66
P08254	Stromelysin-1 (MMP3)	SP	6.11

SecretP: secretome P score, SP: Signal Peptide, MEAN: average heavy/light incorporation ratio.
