# Supplementary material for: CILAIR-Based Secretome Analysis of Obese Visceral and Subcutaneous Adipose Tissues Reveals Distinctive ECM Remodeling and Inflammation Mediators
Source: Sci Rep. 2015 Jul 22;5:12214. doi: 10.1038/srep12214 (PMC4648467; doi:10.1038/srep12214)
Supplement: Supplementary Information [file srep12214-s1.pdf]

SUPPLEMENTARY MATERIAL

CILAIR-BASED SECRETOME ANALYSIS OF OBESE VISCERAL AND SUBCUTANEOUS ADIPOSE TISSUES REVEALS DISTINCTIVE ECM REMODELING AND INFLAMATION MEDIATORS.

Arturo Roca-Rivada, Susana Belen Bravo, Diego Perez-Sotelo, Jana Alonso, Ana Isabel Castro, Ivan Baamonde, Javier Baltar, Felipe F Casanueva, Maria Pardo.

SUPPLEMENTARY TABLE 1

	PROTEIN ID	PROTEIN NAME	Secretome P	Sequence coverage % Cov (95)
VAT	|P31946|1433B_HUMAN	14-3-3 protein beta/alpha	0,352	15,5
	|P63261|ACTG_HUMAN	Actin, cytoplasmic 2	0,505	21,1
	|Q15848|ADIPO_HUMAN	Adiponectin	SP	6,1
	|P01023|A2MG_HUMAN	Alpha-2-macroglobulin	SP	4,2
	|P05067|A4_HUMAN	Amyloid beta A4 protein	SP	1,3
	|P07355|ANXA2_HUMAN	Annexin A2	0,746	8,9
	|P02647|APOA1_HUMAN	Apolipoprotein A-I	SP	19,1
	|P02649|APOE_HUMAN	Apolipoprotein E	SP	1
	|P20160|CAP7_HUMAN	Azurocidin	SP	5,2
	|Q8NE79|POPD1_HUMAN	Blood vessel epicardial substance O	0,565	1
	|P27797|CALR_HUMAN	Calreticulin	SP	11,3
	|O94985|CSTN1_HUMAN	Calsyntenin-1	SP	2,4
	|P00915|CAH1_HUMAN	Carbonic anhydrase 1	0,62	18,0
	|P16870|CBPE_HUMAN	Carboxypeptidase E	SP	1,0
	|Q9UBR2|CATZ_HUMAN	Cathepsin Z	SP	3,3
	|Q03135|CAV1_HUMAN	Caveolin-1	0,5	11,8
	|Q6YHK3|CD109_HUMAN	CD109 antigen	SP	1,3
	|P16070|CD44_HUMAN	CD44 antigen	SP	1,6
	|P43121|MUC18_HUMAN	Cell surface glycoprotein MUC18	SP	4,5
	|Q9P2E5|CHPF2_HUMAN	Chondroitin sulfate glucuronyltransferase	SP	1,0
	|P10909|CLUS_HUMAN	Clusterin	SP	2,7
	|P02452|CO1A1_HUMAN	Collagen alpha-1(I) chain	SP	24,9
	|P02461|CO3A1_HUMAN	Collagen alpha-1(III) chain	SP	16,3
	|P02462|CO4A1_HUMAN	Collagen alpha-1(IV) chain	SP	8,6
	|P20908|CO5A1_HUMAN	Collagen alpha-1(V) chain	SP	2,2
	|Q05707|COEA1_HUMAN	Collagen alpha-1(XIV) chain	SP	4,7
	|P05997|CO5A2_HUMAN	Collagen alpha-2(V) chain	SP	1,0
	|P0C0L5|CO4B_HUMAN	Complement C4-B	SP	1,0
	|P10643|CO7_HUMAN	Complement component C7	SP	1,0
	|P00746|CFAD_HUMAN	Complement factor D (Adipsin)	SP	5,1
	|P08603|CFAH_HUMAN	Complement factor H	SP	1,5
	|P07585|PGS2_HUMAN	Decorin	0,506	10,3
	|Q07507|DERM_HUMAN	Dermatopontin	SP	4,0
	|P53634|CATC_HUMAN	Dipeptidyl peptidase 1	SP	2,4
	|O43184|ADA12_HUMAN	Disintegrin and metalloproteinase domain-containing protein 12	SP	1,3
	|Q13822|ENPP2_HUMAN	Ectonucleotide pyrophosphatase/phosphodiesterase family member 2	SP	1,6
	|Q9Y6C2|EMIL1_HUMAN	EMILIN-1	SP	2,3
	|Q9NZ08|ERAP1_HUMAN	Endoplasmic reticulum aminopeptidase 1	SP	2,8
	|Q9HCU0|CD248_HUMAN	Endosialin	SP	7,9

|P07099|HYEP_HUMAN	Epoxide hydrolase 1	SP	4,0
|P08294|SODE_HUMAN	Extracellular superoxide dismutase [Cu-Zn]	SP	6,7
|P15090|FABP4_HUMAN	Fatty acid-binding protein, adipocyte	0,747	39,4
|P35555|FBN1_HUMAN	Fibrillin-1	SP	0,7
|P02675|FIBB_HUMAN	Fibrinogen beta chain	SP	30,6
|P02679|FIBG_HUMAN	Fibrinogen gamma chain	SP	15,7
|Q06828|FMOD_HUMAN	Fibromodulin	SP	4,5
|P98095|FBLN2_HUMAN	Fibulin-2	SP	0,8
|Q9UBX5|FBLN5_HUMAN	Fibulin-5	SP	5,6
|Q12841|FSTL1_HUMAN	Follistatin-related protein 1	SP	3,2
|P04792|HB1_HUMAN	Heat shock protein beta-1	0,74	4,4
|P02790|HEMO_HUMAN	Hemopexin	SP	2,4
|Q96QV6|H2A1A_HUMAN	Histone H2A type 1-A	0,516	6,9
|Q9BTM1|H2AJ_HUMAN	Histone H2A.J	0,559	12,4
|Q16695|H31T_HUMAN	Histone H3.1t	0,696	8,1
|Q29836|1B67_HUMAN	HLA class I histocompatibility antigen, B-67 alpha chain	SP	3,6
|P01876|IGHA1_HUMAN	Ig alpha-1 chain C region	0,732	7,1
|P01857|IGHG1_HUMAN	Ig gamma-1 chain C region	0,581	11,8
|P01859|IGHG2_HUMAN	Ig gamma-2 chain C region	0,638	11,4
|P01767|HV306_HUMAN	Ig heavy chain V-III region BUT	0,705	9,6
|P01772|HV311_HUMAN	Ig heavy chain V-III region KOL	0,909	12,7
|P01834|IGKC_HUMAN	Ig kappa chain C region	0,659	48,1
|P01703|LV105_HUMAN	Ig lambda chain V-I region NEWM	0,839	16,5
|P0CG06|LAC3_HUMAN	Ig lambda-3 chain C regions	0,783	28,3
|P01871|IGHM_HUMAN	Ig mu chain C region	0,566	5,8
|P04220|MUCB_HUMAN	Ig mu heavy chain disease protein	0,509	4,9
|O14498|ISLR_HUMAN	Immunoglobulin superfamily containing leucine-rich repeat protein	SP	1,9
|P22692|IBP4_HUMAN	Insulin-like growth factor-binding protein 4	SP	7,8
|Q16270|IBP7_HUMAN	Insulin-like growth factor-binding protein 7	SP	7,8
|P05231|IL6_HUMAN	Interleukin-6	SP	8,0
|P10145|IL8_HUMAN	Interleukin-8	SP	16,2
|O75874|IDHC_HUMAN	Isocitrate dehydrogenase [NADP] cytoplasmic	0,547	1,0
|Q76M96-2|CCD80_HUMAN	Isoform 2 of Coiled-coil domain-containing protein 80	SP	4,2
|P23142-4|FBLN1_HUMAN	Isoform C of Fibulin-1	SP	2,9
|P02751-9|FINC_HUMAN	Isoform Fibronectin not containing EIIIA and EIIIB and uses V64 variant of IIICS region of Fibronectin	SP	9,0
|P13611-5|CG2_HUMAN	Isoform Vint of Versican core protein	SP	3,4
|P02788|TRFL_HUMAN	Lactotransferrin	SP	1,7
|O15230|LAMA5_HUMAN	Laminin subunit alpha-5	SP	0,4
|P07942|LAMB1_HUMAN	Laminin subunit beta-1	SP	1,1
|P55268|LAMB2_HUMAN	Laminin subunit beta-2	SP	1,1

|Q99538|LGMN_HUMAN	Legumain	SP	3,9
|P41159|LEP_HUMAN	Leptin	SP	10,8
|P15018|LIF_HUMAN	Leukemia inhibitory factor	SP	8,4
|P30740|ILEU_HUMAN	Leukocyte elastase inhibitor	0,516	3,7
|P18428|LBP_HUMAN	Lipopolysaccharide-binding protein	SP	2,3
|P23141|EST1_HUMAN	Liver carboxylesterase 1	SP	27,0
|P00338|LDHA_HUMAN	L-lactate dehydrogenase A chain	0,549	9,3
|P07195|LDHB_HUMAN	L-lactate dehydrogenase B chain	0,569	6,3
|P51884|LUM_HUMAN	Lumican	SP	44,1
|P09603|CSF1_HUMAN	Macrophage colony-stimulating factor 1	SP	3,2
|Q5VSK2|MRC1L_HUMAN	Macrophage mannose receptor 1-like protein 1	SP	0,8
|P33908|MA1A1_HUMAN	Mannosyl-oligosaccharide 1,2-alpha-mannosidase IA	SP	2,1
|P14780|MMP9_HUMAN	Matrix metalloproteinase-9	SP	2,1
|Q16853|AOC3_HUMAN	Membrane primary amine oxidase	SP	5,9
|P55083|MFAP4_HUMAN	Microfibril-associated glycoprotein 4	SP	1,0
|P05164|PERM_HUMAN	Myeloperoxidase	SP	2,4
|P80188|NGAL_HUMAN	Neutrophil gelatinase-associated lipocalin	SP	7,6
|P14543|NID1_HUMAN	Nidogen-1	SP	6,7
|P26022|PTX3_HUMAN	Pentraxin-related protein PTX3	SP	1,5
|P19021|AMD_HUMAN	Peptidyl-glycine alpha-amidating monooxygenase	SP	1,5
|Q06830|PRDX1_HUMAN	Peroxiredoxin-1	0,528	5,0
|P32119|PRDX2_HUMAN	Peroxiredoxin-2	0,522	9,1
|P30086|PEBP1_HUMAN	Phosphatidylethanolamine-binding protein 1	0,672	17,7
|P55058|PLTP_HUMAN	Phospholipid transfer protein	SP	3,0
|P05155|IC1_HUMAN	Plasma protease C1 inhibitor	SP	5,4
|P05120|PAI2_HUMAN	Plasminogen activator inhibitor 2	0,536	13,4
|P13796|PLSL_HUMAN	Plastin-2	0,502	2,6
|P07602|SAP_HUMAN	Proactivator polypeptide	SP	2,5
|Q96SM3|CPXM1_HUMAN	Probable carboxypeptidase X1	SP	2,0
|Q15084|PDIA6_HUMAN	Protein disulfide-isomerase A6	SP	3,9
|Q6DN03|H2B2C_HUMAN	Putative histone H2B type 2-C	0,575	5,2
|P49788|TIG1_HUMAN	Retinoic acid receptor responder protein 1	SP	6,1
|P02753|RET4_HUMAN	Retinol-binding protein 4	SP	5,0
|P07998|RNAS1_HUMAN	Ribonuclease pancreatic	SP	4,5
|O75326|SEM7A_HUMAN	Semaphorin-7A	SP	2,3
|P02787|TRFE_HUMAN	Serotransferrin	SP	6,4
|P02768|ALBU_HUMAN	Serum albumin	SP	50,6
|P02743|SAMP_HUMAN	Serum amyloid P-component	SP	4,5
|O75368|SH3L1_HUMAN	SH3 domain-binding glutamic acid-rich-like protein	0,758	8,8
|A6NMB1|SIG16_HUMAN	Sialic acid-binding Ig-like lectin 16	SP	1,0
|Q13621|S12A1_HUMAN	Solute carrier family 12 member 1	0,565	1,0

	|Q9BUD6|ON2_HUMAN	Spondin-2	SP	3,6
	|P09238|MMP10_HUMAN	Stromelysin-2	SP	2,5
	|P04179|SODM_HUMAN	Superoxide dismutase [Mn], mitochondrial	0,535	6,3
	|P22105|TENX_HUMAN	Tenascin-X	SP	1,0
	|Q9UKZ4|TEN1_HUMAN	Teneurin-1	0,591	1,0
	|Q8NBS9|TXND5_HUMAN	Thioredoxin domain-containing protein 5	SP	4,2
	|P10646|TFPI1_HUMAN	Tissue factor pathway inhibitor	SP	2,6
	|P48307|TFPI2_HUMAN	Tissue factor pathway inhibitor 2	SP	14,9
	|P60174|TPIS_HUMAN	Triosephosphate isomerase	0,51	22,5
	|Q15661|TRYB1_HUMAN	Tryptase beta-1	SP	1,0
	|P62988|UBIQ_HUMAN	Ubiquitin		17,1
	|Q969H8|CS010_HUMAN	UPF0556 protein C19orf10	SP	5,2
	|P00749|UROK_HUMAN	Urokinase-type plasminogen activator	SP	2,1
	|P08670|VIME_HUMAN	Vimentin	0,512	13,3
	|P07225|PROS_HUMAN	Vitamin K-dependent protein S	SP	1,2
	|P04004|VTNC_HUMAN	Vitronectin	SP	3,1
	**PROTEIN ID**	**PROTEIN NAME**	**Secretome P**	**Sequence coverage**
**SAT**	|P02749|APOH_HUMAN	Beta-2-glycoprotein 1	SP	5,8
	|P20908|CO5A1_HUMAN	Collagen alpha-1(V) chain	SP	1,0
	|P09871|C1S_HUMAN	Complement C1s subcomponent	SP	6,0
	|P08174|DAF_HUMAN	Complement decay-accelerating factor	SP	6,3
	|Q16610|ECM1_HUMAN	Extracellular matrix protein 1	SP	11,3
	|P02671|FIBA_HUMAN	Fibrinogen alpha chain	SP	13,5
	|Q92820|GGH_HUMAN	Gamma-glutamyl hydrolase *	SP	4,4
	|P35052|GPC1_HUMAN	Glypican-1	SP	3,4
	|P00738|HPT_HUMAN	Haptoglobin	SP	4,2
	|P04196|HRG_HUMAN	Histidine-rich glycoprotein	SP	1,7
	|P01860|IGHG3_HUMAN	Ig gamma-3 chain C region	0,544	15,1
	|P01861|IGHG4_HUMAN	Ig gamma-4 chain C region	0,609	4,3
	|P00338-2|LDHA_HUMAN	Isoform 2 of L-lactate dehydrogenase A chain	0,549	6,3
	|P56181-2|NDUV3_HUMAN	Isoform 2 of NADH dehydrogenase [ubiquinone] flavoprotein 3, mitochondrial	0,815	1,0
	|P60174-2|TPIS_HUMAN	Isoform 2 of Triosephosphate isomerase	0,51	11,2
	|Q15063-4|POSTN_HUMAN	Isoform 4 of Periostin	SP	13,1
	|P05164-3|PERM_HUMAN	Isoform A of Myeloperoxidase	SP	4,1
	|Q13822-2|ENPP2_HUMAN	Isoform Alpha of Ectonucleotide pyrophosphatase/phosphodiesterase family member 2	SP	9,9
	|P02751-7|FINC_HUMAN	Isoform Fibronectin containing extra ED-B domain of Fibronectin	SP	10,5
	|O14786|NRP1_HUMAN	Neuropilin-1	SP	1,7
	|P59666|DEF3_HUMAN	Neutrophil defensin 3	SP	19,2

	|P02760|AMBP_HUMAN	Protein AMBP	SP	2,3
	|Q9NS98|SEM3G_HUMAN	Semaphorin-3G	SP	3,1
	|Q9BQG1|SYT3_HUMAN	Synaptotagmin-3	0,618	1,9
	|Q01995|TAGL_HUMAN	Transgelin	0,56	9,5

Supplementary material to Figure 4

Complement C3 western blot

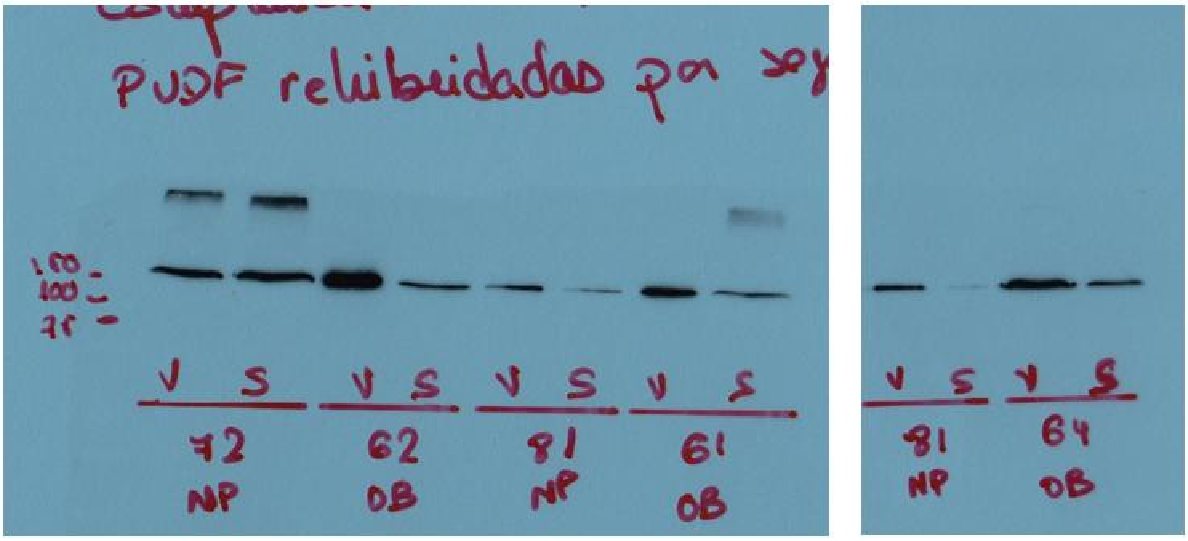

Whole membrane images of Complement C3 western blot from Figure 4A are shown.

Supplementary material to Figure 4

TIMP-1 western blot

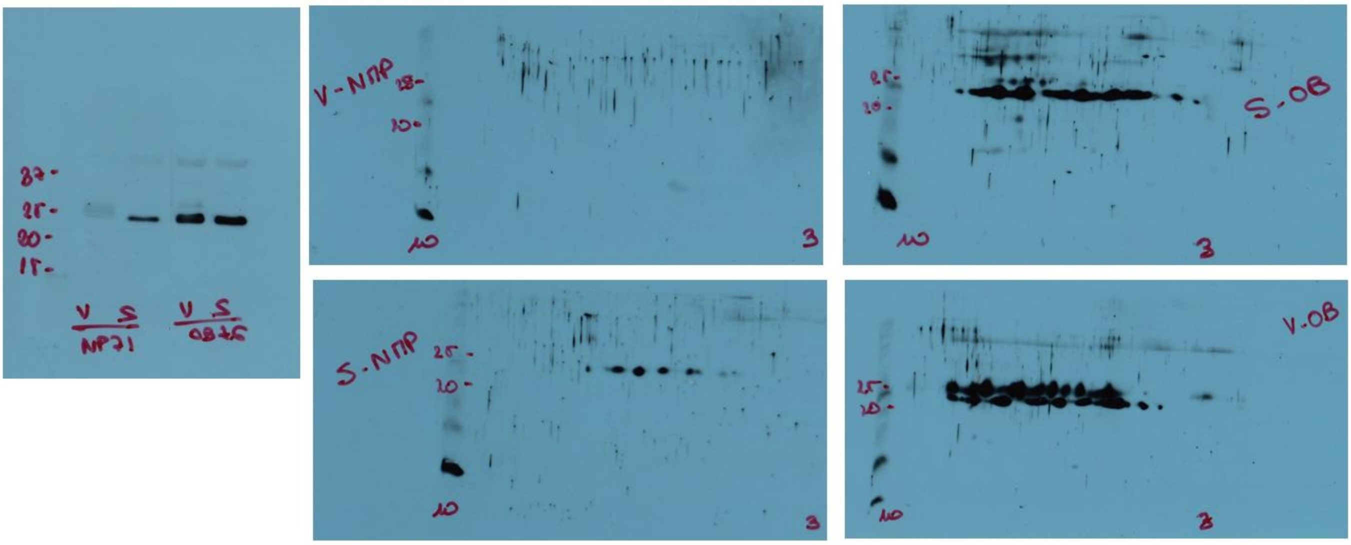

Whole membrane images of 1-D and 2-D TIMP-1 western blots from Figure 4B are shown.
